# Vitamin D and COVID-19: causal factor or bystander?

**DOI:** 10.1136/postgradmedj-2020-139388

**Published:** 2021-01-15

**Authors:** Ching-lung Cheung, Bernard M Y Cheung

**Affiliations:** Department of Pharmacology and Pharmacy, Li Ka Shing Faculty of Medicine, University of Hong Kong, Hong Kong, Hong Kong; Department of Medicine, Li Ka Shing Faculty of Medicine, University of Hong Kong, Hong Kong, Hong Kong

Vitamin D has been attracting a lot of attention because of its potential extraskeletal effects in health and in various diseases such as diabetes, cardiovascular diseases, cancer and autoimmune diseases.^1 2^ Most of these diseases have a common underlying pathogenesis, namely, dysregulated immune function and increased inflammation.^1^

Owing to these immunomodulatory properties, a number of studies have investigated the relationship of vitamin D status with COVID-19. In this issue, Baktash *et al* 2 evaluated the association of vitamin D with COVID-19 status in 105 patients (70 and 35 were COVID-19 positive and negative, respectively), and whether it is associated with clinical outcomes in patients with COVID-19. In brief, the authors concluded that patients with lower vitamin D levels were associated with COVID-19 infection. Among those patients with COVID-19 (N=70), vitamin D deficiency (defined as circulating 25-hydroxyvitamin D ≤30 nmol/L) was associated with higher peak D-dimer level, incidence of non-invasive ventilatory support and dependency unit admission when compared with age-matched vitamin D replete patients (defined as circulating 25-hydroxyvitamin D >30 nmol/L). Thus, this observational study shed light on the potential role of vitamin D as a simple way of countering COVID-19 infection.

Preclinical studies have demonstrated that vitamin D is important in immune function.^3^ It is immunomodulatory by regulating expression of cytokines. Given that elevated inflammation is commonly found in COVID-19, a better regulation of cytokines release may lead to a better prognosis. In addition, vitamin D also induces expression of antimicrobial peptides (such as β-defensin and cathelicidin), which are known to act against enveloped viruses, of which the SARS-CoV-2 is one. These may be the mechanisms explaining the potential protective effect of vitamin D against COVID-19.

However, it should be noted that causality cannot be inferred in an observational study. Vitamin D insufficiency or deficiency might simply be a marker of frailty or physically inactivity, both of which are known to be associated with adverse clinical outcomes, including increased risk of infection. The lesson we have learnt from previous vitamin D studies is that randomised controlled trials are still necessary for demonstrating the clinical therapeutic value of vitamin D. For example, higher circulating vitamin D levels have been shown to be associated with reduced risk of cancer and cardiovascular diseases; but in the VITAL trial involving 25 871 participants, supplementation of a daily dose of 2000 IU did not lower the risk of either invasive cancer or cardiovascular events compared with placebo.^4^ Notably, the lack of an observable effect of vitamin D on clinical outcomes in trials is multifactorial, and could be due to a genuine null effect of vitamin D on the respective clinical outcomes, or it could be due to the length of follow-up, baseline vitamin D status, dose and sample size. While it seems reasonable to assume that a higher dose of vitamin D might result in a better therapeutic effect, we should be aware of the potential non-linear effect of vitamin D on health outcomes, including skeletal ones. A recent RCT showed that high-dose vitamin D supplementation (10 000 IU/day) had a potential harmful effect on bone mineral density and microarchitecture,^5^ which is opposite to the popular concept that vitamin D is good for bone, although whether the harm was caused by vitamin D or mediated through mineral metabolism^6^ is unknown. Thus, a well-designed RCT of vitamin D is not straightforward and requires careful planning.

Given that vitamin D deficiency or insufficiency are highly prevalent globally,^7 8^ there is no harm in recommending optimal sunlight exposure or vitamin D supplementation to the general public from the nutritional perspective. However, it may be premature to make any claims on the indication of vitamin D for COVID-19. Blind faith in taking vitamin D to prevent COVID-19 may give a false sense of invulnerability and increase the risk of infection and delay seeking medical diagnosis and treatment. Studies such as the one by Baktash *et al* 2 highlight a potential simple weapon against COVID-19, and pave the way for carefully designed RCTs to establish the efficacy and safety of vitamin D in managing COVID-19 infections.
